# Automated Analysis of Drawing Process to Estimate Global Cognition in Older Adults: Preliminary International Validation on the US and Japan Data Sets

**DOI:** 10.2196/37014

**Published:** 2022-05-05

**Authors:** Yasunori Yamada, Kaoru Shinkawa, Masatomo Kobayashi, Varsha D Badal, Danielle Glorioso, Ellen E Lee, Rebecca Daly, Camille Nebeker, Elizabeth W Twamley, Colin Depp, Miyuki Nemoto, Kiyotaka Nemoto, Ho-Cheol Kim, Tetsuaki Arai, Dilip V Jeste

**Affiliations:** 1 Digital Health IBM Research Tokyo Japan; 2 Department of Psychiatry University of California San Diego La Jolla, CA United States; 3 Sam and Rose Stein Institute for Research on Aging University of California San Diego La Jolla, CA United States; 4 VA San Diego Healthcare System San Diego, CA United States; 5 Department of Family Medicine and Public Health University of California San Diego La Jolla, CA United States; 6 Department of Psychiatry, Division of Clinical Medicine, Faculty of Medicine University of Tsukuba Ibaraki Japan; 7 AI and Cognitive Software IBM Almaden Research Center San Jose, CA United States; 8 Department of Neurosciences University of California San Diego La Jolla, CA United States

**Keywords:** tablet, behavior analysis, digital biomarkers, digital health, motor control, cognitive impairment, dementia, machine learning, multicohort, multination

## Abstract

**Background:**

With the aging of populations worldwide, early detection of cognitive impairments has become a research and clinical priority, particularly to enable preventive intervention for dementia. Automated analysis of the drawing process has been studied as a promising means for lightweight, self-administered cognitive assessment. However, this approach has not been sufficiently tested for its applicability across populations.

**Objective:**

The aim of this study was to evaluate the applicability of automated analysis of the drawing process for estimating global cognition in community-dwelling older adults across populations in different nations.

**Methods:**

We collected drawing data with a digital tablet, along with Montreal Cognitive Assessment (MoCA) scores for assessment of global cognition, from 92 community-dwelling older adults in the United States and Japan. We automatically extracted 6 drawing features that characterize the drawing process in terms of the drawing speed, pauses between drawings, pen pressure, and pen inclinations. We then investigated the association between the drawing features and MoCA scores through correlation and machine learning–based regression analyses.

**Results:**

We found that, with low MoCA scores, there tended to be higher variability in the drawing speed, a higher pause:drawing duration ratio, and lower variability in the pen’s horizontal inclination in both the US and Japan data sets. A machine learning model that used drawing features to estimate MoCA scores demonstrated its capability to generalize from the US dataset to the Japan dataset (*R^2^*=0.35; permutation test, *P*<.001).

**Conclusions:**

This study presents initial empirical evidence of the capability of automated analysis of the drawing process as an estimator of global cognition that is applicable across populations. Our results suggest that such automated analysis may enable the development of a practical tool for international use in self-administered, automated cognitive assessment.

## Introduction

With the aging of populations worldwide, early detection of cognitive impairments has become a research and clinical priority. In particular, early identification of prodromal dementia is essential for providing secondary prevention and disease-modifying treatments [1-4]. The cognitive screening tests most commonly used by clinicians are the Mini-Mental State Examination (MMSE) [5] and the Montreal Cognitive Assessment (MoCA) [6]. Both tests are designed to assess global cognition, and validated cutoff scores are used for detecting impairment [7,8]. One limitation of these tests is that they require administration by trained professionals. According to the World Alzheimer Report published in 2021 [1], 83% of clinicians reported that the COVID-19 pandemic has delayed access to cognitive screening tests. Consequently, self-administered, automated assessment may be more important in situations, like the current COVID-19 pandemic, that impose limitations on in-person evaluation in a clinical setting. Another limitation of these tests is related to issues with their use in multilingual populations, such as cross-linguistic artifacts in translation [1,9,10]. Recently, several nonlinguistic cognitive tests have been investigated to overcome the influence of language differences by mitigating the need for translation [11,12]. In sum, there is a clear need to develop a self-administered, automated assessment tool that can be used internationally, which would greatly increase the accessibility of screening in a variety of settings and populations. This would be particularly important for removing barriers to diagnosis and mitigating the gap between countries in the diagnostic coverage—the rate of diagnosis of dementia was estimated to be only 25% worldwide, with less than 10% in low- and middle-income countries [1].

Drawing ability is a promising means for developing such an automated cognitive assessment tool. Drawing tests have been widely used for screening cognitive impairments and dementia (eg, trail making [13] and clock drawing [14]), and automated analysis of the drawing process has shown that features characterizing the drawing process are sensitive to cognitive impairments and diagnoses of dementia [15-18]. For example, reduction in the drawing speed and increases in its variability, as well as increased pauses between drawing motions, have been reported as statistically significant features for assessment of impaired global cognition [19,20], as well as for detecting Alzheimer disease (AD) and mild cognitive impairment (MCI) [21-24]. Machine learning models based on these drawing features have succeeded in estimating measures of global cognition [25,26] and classifying AD, MCI, and control individuals [23-25,27]. However, there has been little evidence of the capability of automated analysis of the drawing process for assessment of cognitive performance across different populations, even though applicability across the intended populations is a requirement for machine learning–based health care tools, including those for screening of dementia [1,28,29].

In this study, we evaluated the applicability of automated analysis of the drawing process for estimating global cognition in community-dwelling older adults across populations in different nations. Specifically, we collected drawing data with a digital tablet, along with MoCA scores for assessing global cognition, from community-dwelling older adults in the United States and Japan. We then investigated the associations between the MoCA scores and drawing features across the 2 data sets. Finally, we built a machine learning model that used the drawing features to estimate MoCA scores, and we evaluated the model’s generalizability from the US data set to the Japan data set.

## Methods

### Ethical Review

The study was approved by the University of California San Diego Human Research Protections Program (HRPP; project number 170466) and the Ethics Committee of the University of Tsukuba Hospital (H29-065). All participants provided written consent to participate in the study after the procedures of the study had been fully explained.

### Participants

The participants were community-dwelling older adults recruited in San Diego County, California and in Ibaraki prefecture, Japan. For the US data set, the participants were residents of the independent living sector of a continuing-care senior housing community and were recruited through short presentations using an HRPP-approved script and flyer. For the Japan data set, the participants were individuals recruited through local recruiting agencies or community advertisements in accordance with the approved protocol. Both data sets represented subsets of larger cohort studies [24,30]. The participant selection criteria were as follows: (1) English-speaking (for the United States) or Japanese-speaking (for Japan) individuals ≥65 years old, (2) completion of the MoCA, (3) no known diagnosis of dementia, and (4) no other diseases or disabilities that would interfere with the collection of drawing data.

Table 1 summarizes the participants’ characteristics. We collected and analyzed drawing data and MoCA scores from a total of 92 community-dwelling older adults in the United States and Japan. The US data set included 55 participants aged 67-98 years (female: 39/55, 71%; age, mean 83.4, SD 6.9 years). The Japan data set included 37 participants aged 65-80 years (female: 19/37, 51%; age: mean 73.3, SD 4.5 years). Regarding the demographics, the proportion of female participants did not differ statistically between the 2 data sets (*χ^2^*_1_=3.63, *P*=.06), while the age and years of education were higher in the US data set than in the Japan data set (age: *t*_90_=7.79, *P*<.001; years of education: *t*_90_=5.25, *P*<.001).

**Table 1:** Participants’ characteristics (n=92).

Characteristics	United States (n=55)	Japan (n=37)	*P* value
Age (years), mean (SD)	83.4 (6.9)	73.3 (4.5)	<.001^a^
Sex (female), n (%)	39 (71)	19 (51)	.06^b^
Education (years), mean (SD)	16.3 (2.3)	13.8 (2.0)	<.001^a^
Montreal Cognitive Assessment^c^, mean (SD)	24.4 (3.2)	24.4 (2.6)	.98^a^
Trail Making Test part B time (seconds), mean (SD)	131.9 (65.1)^d^	96.9 (50.1)^d^	.008^a^
Trail Making Test part B errors, mean (SD)	1.7 (2.5)^d^	0.9 (1.5)^d^	.07^a^

^a^Compared using 2-sided *t* tests.

^b^Compared using a chi square test.

^c^Total possible score ranges from 0 to 30.

^d^Data were missing for 1 participant because of incomplete trials.

### Data Analysis

All participants performed the Trail Making Test part B (TMT-B) [13] and MoCA. The TMT-B drawing data were collected using a Wacom Cintiq Pro 16 tablet (sampling rate: 180 Hz; drawing area size: 252 × 186 mm; pen pressure levels: 8192; pen inclination resolution: 1 degree) and custom Windows software that we developed. The software was written in the C# language and was used to capture raw drawing data from the tablet via the Wacom Wintab .NET library (version: 1.2). The raw data consisted of a time series of the pen tip's x- and y-coordinates, the pen pressure, the pen's horizontal and vertical inclinations, and the distance of the pen tip from the drawing surface. All data were captured at the tablet's sampling rate.

The TMT-B was selected as a representative cognitive task that involves drawing motions and is commonly used in clinical practice for screening AD and MCI [31,32]. It requires participants to draw lines that alternately connect a total of 25 numbers and letters in their respective sequences [13]. For the MoCA, we used the original paper-and-pencil version [6] for the US participants and its Japanese version [33] for the Japan participants. The total possible score on the MoCA ranges from 0 to 30, where lower scores indicate lower global cognition. Both TMT-B and the MoCA were administered by neuropsychologists or trained study staff who were blind to the study hypothesis during data collection. The US data set was collected between May 2019 and January 2020. The Japan data set was collected between December 2018 and May 2019.

Next, we extracted drawing features from the drawing data and examined their associations with the MoCA scores. Specifically, we investigated the following 6 automatically extracted drawing features: the drawing speed and its variability, the pressure variability, the variabilities of the pen’s horizontal and vertical inclinations, and the pause:drawing duration ratio. These features were selected because they have been reported as significant indicators of changes in cognitive or motor functions [15,16,24,34]. The drawing speed represented the speed of the pen tip on the surface during drawing motions. The drawing speed variability was calculated using the coefficient of variation to remove the influence of the absolute value, as the drawing speed itself was also a feature. For the pressure variability, we used the median absolute deviation, which is more robust against outliers than the standard deviation. In contrast, the variabilities of the pen’s horizontal and vertical inclinations were calculated using standard deviations. The pause:drawing duration ratio was defined as the ratio of the total duration of pauses between drawing motions (ie, between strokes and within a stroke) and the total duration of drawing motions on the surface. Pauses within a stroke were detected when the pen tip remained inside a 0.25-mm radius on the drawing surface for more than 100 milliseconds.

To investigate the associations of each drawing feature with the MoCA scores, Pearson correlation coefficients were computed after controlling for the age, sex, and years of education for the entire data set and for the US and Japan data sets separately. The 3 sociodemographic variables were considered as covariates, because they have been suggested to affect performance on cognitive screening tests, including the MoCA [35]. The following Python 3.8 libraries were used for the correlation analysis: pandas (version 1.2.4), NumPy (version 1.20.1), SciPy (version 1.6.2), and pingouin (version 0.4.0).

**Figure 1:**
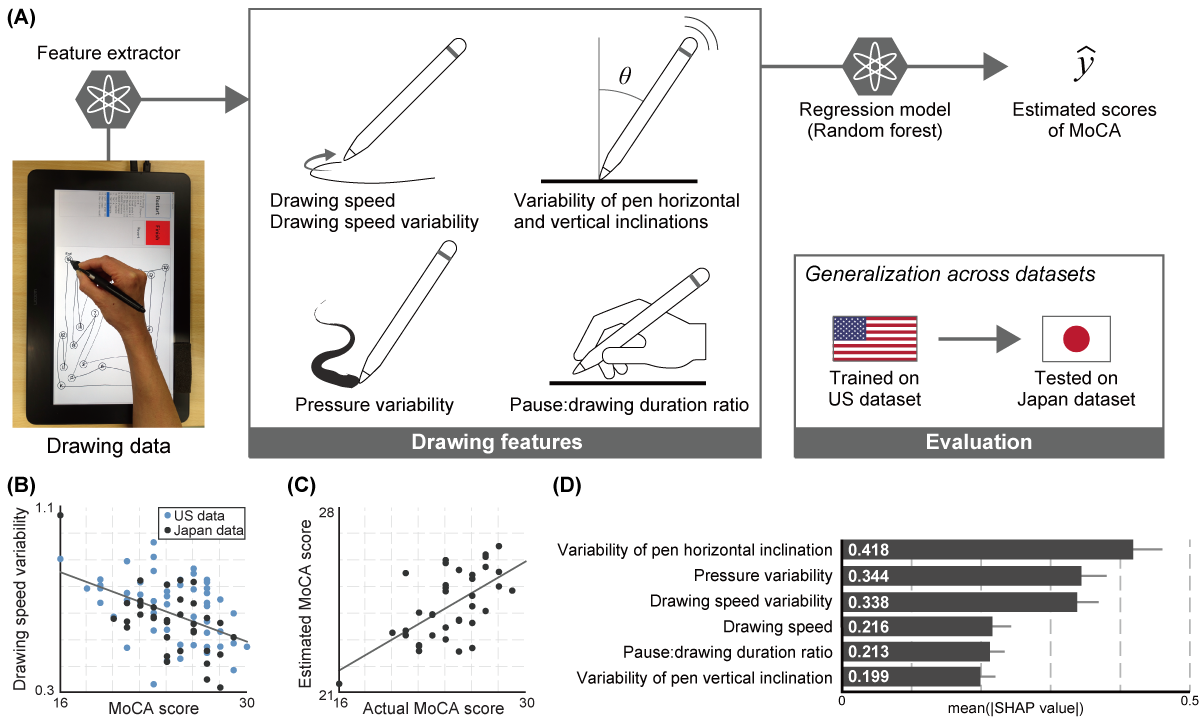
Study overview: (A) workflow of the automated analysis in which drawing data were collected with a digitizing tablet and pen, 6 drawing features were extracted from the drawing data, and a regression model for estimating Montreal Cognitive Assessment (MoCA) scores was trained on the US data set and tested on the Japan data set; (B) plot of the drawing speed variability with respect to the MoCA score for the US and Japan data sets, in which each point represents 1 participant and the solid line represents the regression line for the combined data set; (C) plot of the estimated and actual MoCA scores in the Japan data set, in which each point represents 1 participant and the solid line represents the regression line; (D) comparison of the features’ importance with standard deviations, as assessed via the mean absolute Shapley Additive Explanations (SHAP) values.

We also developed a supervised machine learning model that used drawing features to estimate MoCA scores, and we then evaluated the model’s applicability across data sets. The analysis workflow is illustrated in Figure 1A. Specifically, the model was trained on the US data set and tested on the Japan data set. For the machine learning model, we used the random forest algorithm to capture nonlinear relationships, given that nonlinear interactions between drawing features and cognitive impairments were observed in previous studies [23,24]. The random forest hyperparameters in this study were as follows: search range of 2, 3, and 4 for the maximum tree depth; 2, 3, 4, and 6 for the maximum number of features; 1.0, 0.75, and 0.5 for the proportion of the maximum number of samples to train each base regressor; and 2, 3, 4, and 5 for the minimum number of samples required at a leaf node. The number of trees was set to 500, and all other parameters were kept at their default values. The hyperparameters were tuned through 10-fold cross-validation within the training data set. We statistically evaluated the observed performance through permutation testing (1000 iterations) by randomizing the MoCA scores. To better interpret the results, the importance of each feature in the resultant model was also evaluated using the Shapley Additive Explanations (SHAP) method [36]. Specifically, we compared the mean absolute SHAP values of each feature. The following Python 3.8 libraries were used to perform the machine learning analysis: scikit-learn (version 0.23.2) and SHAP (version 0.40.0).

## Results

The mean MoCA score was 24.4 (SD 3.0; range for participants: 16-30; possible range: 0-30), and the scores did not differ statistically between the 2 data sets (*t*_90_= 0.02, *P*=.99; Table 1). For the collection of drawing data, each session took an average of 119.7 (SD 64.6) seconds per participant. The mean TMT-B time and number of errors were 117.9 (SD 61.7) seconds and 1.4 (SD 2.2), respectively. The TMT-B time was longer in the US data set (*t*_88_=2.72, *P*=.008), while the number of errors did not differ statistically between the 2 data sets (*t*_88_=1.82, *P*=.07). Two participants (US: 1; Japan: 1) could not complete the TMT-B trial. To include them in the analysis, we used features extracted from their partial drawing data.

For the correlation analysis between the MoCA scores and each drawing feature in the entire data set, we found that 4 of the 6 features were significantly associated after controlling for age, sex, and years of education (absolute Pearson *r*=0.33-0.49, *P*≤.002; see Figure 1B for a correlation example and Table 2 for the full list). With lower MoCA scores, there tended to be higher variability in the drawing speed and pen pressure, a higher pause:drawing duration ratio, and lower variability in the pen’s horizontal inclination. As listed in Table 2, these tendencies were also observed when the 2 data sets were each analyzed separately. After correction for multiple comparisons, all the statistically significant correlations remained for the entire data set and the Japan data set (Benjamini-Hochberg adjusted *P*<.05), whereas those for the US data set lost significance (Benjamini-Hochberg adjusted *P*>.05).

The random forest model trained on the US data set could estimate MoCA scores from drawing features for the Japan data set with an *R^2^* of 0.35 (Pearson *r* of 0.61, mean absolute error of 1.75, and root-mean-square error of 2.12; permutation test, *P*<.001; Figure 1C). Regarding the importance of each feature in the model, as indicated by the SHAP values, the variability of the pen’s horizontal inclination had the highest importance, followed by the pressure variability and the drawing speed variability (Figure 1D).

**Table 2:** Partial correlations between drawing features and Montreal Cognitive Assessment (MoCA) scores after controlling for age, sex, and years of education.

Drawing features	All (n=92)		United States (n=55)		Japan (n=37)	
	Pearson *r* (95% CI)	*P* value	Pearson *r* (95% CI)	*P* value	Pearson *r* (95% CI)	*P* value
Drawing speed	0.08 (−0.14 to 0.28)	.48	0.09 (−0.19 to 0.35)	.53	0.14 (−0.21 to 0.45)	.44
Drawing speed variability	−0.42 (−0.58 to −0.23)	<.001	−0.33 (−0.55 to −0.06)	.02	−0.58 (−0.77 to −0.31)	<.001
Pause:drawing duration ratio	−0.49 (−0.63 to −0.31)	<.001	−0.32 (−0.55 to −0.06)	.02	−0.73 (−0.86 to −0.53)	<.001
Pressure variability	−0.34 (−0.51 to −0.14)	.001	−0.26 (−0.49 to 0.02)	.07	−0.49 (−0.71 to −0.18)	.003
Variability of pen's horizontal inclination	0.33 (0.13 to 0.50)	.002	0.30 (0.03 to 0.53)	.03	0.38 (0.04 to 0.63)	.03
Variability of pen's vertical inclination	0.17 (−0.04 to 0.37)	.11	0.26 (–0.01 to 0.50)	.06	0.16 (–0.19 to 0.47)	.37

## Discussion

### Principal Findings

We collected drawing data from 92 community-dwelling older adults in the United States and Japan, and we investigated the associations between features characterizing the drawing process and global cognition as assessed by MoCA. We obtained 2 main findings, as follows. First, we found drawing features that showed consistent trends with respect to the changes in MoCA scores across the US and Japan data sets. Specifically, with low MoCA scores, there tended to be higher variability in the drawing speed, a higher pause:drawing duration ratio, and lower variability in the pen’s horizontal inclination. Our second finding was that the automated machine learning model trained on the drawing data in the US data set could estimate the MoCA scores for the Japan data set with an *R^2^* of 0.35, particularly by leveraging variability-related features. We used drawing data from the TMT-B task in this study, but other types of drawing tasks may have a similar capability. For example, a previous study showed that MoCA scores could be estimated by using pause- and speed-based features from a clock drawing task [26], although the method's applicability across populations was not evaluated. The use of 2 or more tasks will be a promising area of future research for more reliable estimation of global cognition.

Regarding the correlations of drawing features with MoCA scores across the US and Japan data sets, the correlations persisted even after controlling for age, sex, and years of education. In post hoc power analysis, the power exceeded 0.90 with a significance level of .05 (2-sided). The trends were consistent with those observed in previous studies with individuals with impaired global cognition [19,20] or patients with AD or MCI [21-24]. One of our contributions lies in demonstrating consistent trends between drawing features and clinical cognitive scores across 2 different populations by using the same protocol. It is especially notable that the pause:drawing duration ratio and the drawing speed variability have been reported as representative features for use in AD or MCI screening models based on automated analysis of the drawing process [23,24]. To our knowledge, the models in those previous studies were not tested for their applicability across different populations, but our results suggest that these drawing features may help with the application of screening models across populations for international use.

We have presented preliminary evidence suggesting that automated analysis of the drawing process for estimation of global cognition can be applied across populations. We trained the machine learning model on drawing data in the US data set, and we then evaluated its performance on unseen drawing data in the Japan data set. In this context, the model could estimate MoCA scores with an *R^2^* of 0.35 (Pearson *r* of 0.61 and root-mean-square error of 2.12). Previous studies investigated models that used a single data set to estimate global cognition from the characteristics of drawing or other types of behaviors such as speech. The performance results for those models included a Pearson correlation coefficient of 0.55 for MoCA on a model using drawing features [26] and a root-mean-square error of 3.74 for MMSE on the best model using speech features in a competition [37]. Our model outperformed those recent results, although there are notable methodological differences in terms of the evaluation method and the sample size, for example. Our model’s improved performance might have derived from the use of variability-related features, given that they were ranked as the most important features in our model. Variability-related features in drawing have recently been suggested as a potential marker for motor control deterioration in dementia [19,38,39], but they have rarely been used for estimating cognitive function, and they have not been tested across populations. Our results thus suggest that variability-related features in drawing may be a key behavioral marker for automatic assessment of global cognition across different populations.

With the aging of populations worldwide, there is a growing interest in using digital technology to assess cognitive function in nonclinical settings like the home for early detection of dementia [1]. Examples of such research include approaches using computerized cognitive tests [29,40-42] and using behavioral data such as drawing, speech, and gait data [24,32,43-45]. In either approach, a major challenge is to make the tool suitable for multinational and multilingual populations [1]. In this context, our results suggest that automated analysis of the drawing process may offer a promising approach for developing such a tool for international use.

Furthermore, the approach using behavioral data is expected to support future efforts toward the development of continuous, passive monitoring tools for early detection of dementia from data that can be collected in everyday life [43,45]. For example, multiple studies have demonstrated the feasibility of detecting cognitive impairments by using daily walking behavior collected from accelerometer sensors in a free-living setting [46-48] and by using daily conversational speech data [49-52]. To our knowledge, no study has investigated the associations of cognitive impairments with daily drawing data that are collected passively in a free-living setting. However, drawing may be a promising behavioral modality for reliable estimation of cognitive impairments: It is a common activity in everyday life, and drawing data can be easily and robustly collected with a commercial-grade device.

Regarding the device used for drawing data collection, previous studies have shown the usefulness of a range of devices, including a mobile tablet with a stylus [53-57], a smart pad [58], and a digital pen [23,26,38]; accordingly, our findings may be applicable to those devices as well. All such devices commonly allow capture of x-and y-coordinates and pressure data at similar sampling rates, and previous studies reported similar associations of pause-, speed-, and pressure-based features with cognitive measures. In a future study, as pen inclination data are not always available, we will need to examine whether a combination of other available data can achieve performance comparable to that of our model. Furthermore, the variability of the device placement (eg, holding the tablet with the nondominant hand) can affect the drawing performance in free-living settings. We will thus need further research in situ for the development of realistic applications.

### Limitations

This study had several limitations. First, it was limited in terms of the numbers of participants, drawing tasks, and data sets. Our findings were based on drawing data from a single task, and the applicability to other types of drawing data thus remains unexplored. In addition, the international applicability of our model was only evaluated between 2 data sets, and the details of how the model performance is influenced by cultural differences have not been thoroughly investigated. Together, our findings have yet to be confirmed with larger samples that provide cross-cultural insights. Second, we did not investigate the participants' sensory and physical functions (eg, eyesight, grip strength), even though those functions might affect drawing performance. Moreover, other residual confounders might exist. Third, the drawing data were collected in a laboratory setting with a tester; accordingly, a future study will need to establish the validity of fully self-administered tasks. Finally, further research will also be needed to obtain a mechanistic understanding of how drawing features relate to the neural changes underlying cognitive impairments.

### Conclusions

In summary, we have presented empirical evidence of the capability of automated analysis of the drawing process as an estimator of global cognition that is applicable across populations. Although no causality could be inferred from our results with cross-sectional data, the results nevertheless suggest that automated analysis of the drawing process could be a practical tool for international use in automated cognitive assessment. Consequently, this approach may help lower the barrier to early detection of cognitive impairments in a variety of settings and populations.

## Abbreviations

AD: Alzheimer disease
HRPP: Human Research Protections Program
MCI: mild cognitive impairment
MMSE: Mini-Mental State Examination
MoCA: Montreal Cognitive Assessment
SHAP: Shapley Additive Explanations
TMT-B: Trail Making Test part B
UCSD: University of California San Diego

## References

[1] Gauthier S, Rosa-Neto P, Morais J, Webster C (2021). World Alzheimer Report 2021. Alzheimer's Disease International.

[2] Livingston G, Huntley J, Sommerlad A, Ames D, Ballard C, Banerjee S, Brayne C, Burns A, Cohen-Mansfield J, Cooper C, Costafreda SG, Dias A, Fox N, Gitlin LN, Howard R, Kales HC, Kivimäki M, Larson EB, Ogunniyi A, Orgeta V, Ritchie K, Rockwood K, Sampson EL, Samus Q, Schneider LS, Selbæk G, Teri L, Mukadam N (2020). Dementia prevention, intervention, and care: 2020 report of the Lancet Commission. The Lancet.

[3] Petersen RC, Lopez O, Armstrong MJ, Getchius TSD, Ganguli M, Gloss D, Gronseth GS, Marson D, Pringsheim T, Day GS, Sager M, Stevens J, Rae-Grant A (2018). Practice guideline update summary: Mild cognitive impairment: Report of the Guideline Development, Dissemination, and Implementation Subcommittee of the American Academy of Neurology. Neurology.

[4] Rasmussen J, Langerman H (2019). Alzheimer's disease - why we need early diagnosis. Degener Neurol Neuromuscul Dis.

[5] Folstein MF, Folstein SE, McHugh PR (1975). "Mini-mental state". A practical method for grading the cognitive state of patients for the clinician. J Psychiatr Res.

[6] Nasreddine ZS, Phillips NA, Bédirian V, Charbonneau S, Whitehead V, Collin I, Cummings JL, Chertkow H (2005). The Montreal Cognitive Assessment, MoCA: a brief screening tool for mild cognitive impairment. J Am Geriatr Soc.

[7] Tombaugh TN, McIntyre NJ (1992). The mini-mental state examination: a comprehensive review. J Am Geriatr Soc.

[8] Carson N, Leach L, Murphy KJ (2018). A re-examination of Montreal Cognitive Assessment (MoCA) cutoff scores. Int J Geriatr Psychiatry.

[9] Ramirez M, Teresi JA, Holmes D, Gurland B, Lantigua R (2006). Differential item functioning (DIF) and the Mini-Mental State Examination (MMSE). Overview, sample, and issues of translation. Med Care.

[10] He J, van de Vijver F (2012). Bias and equivalence in cross-cultural research. Online Readings in Psychology and Culture.

[11] Kandiah N, Zhang A, Bautista DC, Silva E, Ting SKS, Ng A, Assam P (2016). Early detection of dementia in multilingual populations: Visual Cognitive Assessment Test (VCAT). J Neurol Neurosurg Psychiatry.

[12] Goudsmit M, Uysal-Bozkir Ö, Parlevliet JL, van Campen JPCM, de Rooij SE, Schmand B (2017). The Cross-Cultural Dementia Screening (CCD): A new neuropsychological screening instrument for dementia in elderly immigrants. J Clin Exp Neuropsychol.

[13] Bowie CR, Harvey PD (2006). Administration and interpretation of the Trail Making Test. Nat Protoc.

[14] Mainland BJ, Amodeo S, Shulman KI (2014). Multiple clock drawing scoring systems: simpler is better. Int J Geriatr Psychiatry.

[15] Impedovo D, Pirlo G (2019). Dynamic handwriting analysis for the assessment of neurodegenerative diseases: a pattern recognition perspective. IEEE Rev. Biomed. Eng.

[16] Vessio G (2019). Dynamic handwriting analysis for neurodegenerative disease assessment: a literary review. Applied Sciences.

[17] De Stefano C, Fontanella F, Impedovo D, Pirlo G, Scotto di Freca A (2019). Handwriting analysis to support neurodegenerative diseases diagnosis: A review. Pattern Recognition Letters.

[18] Chan JYC, Bat BKK, Wong A, Chan TK, Huo Z, Yip BHK, Kowk TCY, Tsoi KKF (2021). Evaluation of digital drawing tests and paper-and-pencil drawing tests for the screening of mild cognitive impairment and dementia: a systematic review and meta-analysis of diagnostic studies. Neuropsychol Rev.

[19] Schröter A, Mergl R, Bürger K, Hampel H, Möller HJ, Hegerl U (2003). Kinematic analysis of handwriting movements in patients with Alzheimer's disease, mild cognitive impairment, depression and healthy subjects. Dement Geriatr Cogn Disord.

[20] Kawa J, Bednorz A, Stępień P, Derejczyk J, Bugdol M (2017). Spatial and dynamical handwriting analysis in mild cognitive impairment. Comput Biol Med.

[21] Werner P, Rosenblum S, Bar-On G, Heinik J, Korczyn A (2006). Handwriting process variables discriminating mild Alzheimer's disease and mild cognitive impairment. J Gerontol B Psychol Sci Soc Sci.

[22] Yan JH, Rountree S, Massman P, Doody RS, Li H (2008). Alzheimer's disease and mild cognitive impairment deteriorate fine movement control. J Psychiatr Res.

[23] Müller S, Herde L, Preische O, Zeller A, Heymann P, Robens S, Elbing U, Laske C (2019). Diagnostic value of digital clock drawing test in comparison with CERAD neuropsychological battery total score for discrimination of patients in the early course of Alzheimer's disease from healthy individuals. Sci Rep.

[24] Yamada Y, Shinkawa K, Kobayashi M, Caggiano V, Nemoto M, Nemoto K, Arai T (2021). Combining multimodal behavioral data of gait, speech, and drawing for classification of Alzheimer’s disease and mild cognitive impairment. JAD.

[25] Ishikawa T, Nemoto M, Nemoto K, Takeuchi T, Numata Y, Watanabe R, Tsukada E, Ota M, Higashi S, Arai T, Yamada Y (2019). Handwriting features of multiple drawing tests for early detection of Alzheimer's disease: a preliminary result. Stud Health Technol Inform.

[26] Souillard-Mandar W, Penney D, Schaible B, Pascual-Leone A, Au R, Davis R (2021). DCTclock: Clinically-interpretable and automated artificial intelligence analysis of drawing behavior for capturing cognition. Front Digit Health.

[27] Garre-Olmo J, Faúndez-Zanuy M, López-de-Ipiña K, Calvó-Perxas L, Turró-Garriga O (2017). Kinematic and pressure features of handwriting and drawing: preliminary results between patients with mild cognitive impairment, Alzheimer disease and healthy controls. Curr Alzheimer Res.

[28] (2021). Good Machine Learning Practice for Medical Device Development: Guiding Principles. Food & Drug Administration.

[29] Staffaroni AM, Tsoy E, Taylor J, Boxer AL, Possin KL (2020). Digital cognitive assessments for dementia: digital assessments may enhance the efficiency of evaluations in neurology and other clinics. Pract Neurol (Fort Wash Pa).

[30] Jeste DV, Glorioso D, Lee EE, Daly R, Graham S, Liu J, Paredes AM, Nebeker C, Tu XM, Twamley EW, Van Patten R, Yamada Y, Depp C, Kim H (2019). Study of independent living residents of a continuing care senior housing community: sociodemographic and clinical associations of cognitive, physical, and mental health. Am J Geriatr Psychiatry.

[31] Soukup VM, Ingram F, Grady JJ, Schiess MC (1998). Trail Making Test: issues in normative data selection. Appl Neuropsychol.

[32] Sánchez-Cubillo I, Periáñez J, Adrover-Roig D, Rodríguez-Sánchez J, Ríos-Lago M, Tirapu J, Barcelo F (2009). Construct validity of the Trail Making Test: Role of task-switching, working memory, inhibition/interference control, and visuomotor abilities. J Int Neuropsychol Soc.

[33] Fujiwara Y, Suzuki H, Yasunaga M, Sugiyama M, Ijuin M, Sakuma N, Inagaki H, Iwasa H, Ura C, Yatomi N, Ishii K, Tokumaru AM, Homma A, Nasreddine Z, Shinkai S (2010). Brief screening tool for mild cognitive impairment in older Japanese: validation of the Japanese version of the Montreal Cognitive Assessment. Geriatr Gerontol Int.

[34] Asselborn T, Gargot T, Kidziński Ł, Johal W, Cohen D, Jolly C, Dillenbourg P (2018). Automated human-level diagnosis of dysgraphia using a consumer tablet. NPJ Digit Med.

[35] Larouche E, Tremblay M, Potvin O, Laforest S, Bergeron D, Laforce R, Monetta L, Boucher L, Tremblay P, Belleville S, Lorrain D, Gagnon J, Gosselin N, Castellano C, Cunnane SC, Macoir J, Hudon C (2016). Normative data for the Montreal Cognitive Assessment in middle-aged and elderly Quebec-French people. Arch Clin Neuropsychol.

[36] Lundberg S, Lee SI (2017). A Unified Approach to Interpreting Model Predictions.

[37] Syed ZS, Syed MSS, Lech M, Pirogova E (2021). Automated recognition of Alzheimer’s dementia using bag-of-deep-features and model ensembling. IEEE Access.

[38] Davoudi A, Dion C, Amini S, Tighe PJ, Price CC, Libon DJ, Rashidi P (2021). Classifying non-dementia and Alzheimer’s disease/vascular dementia patients using kinematic, time-based, and visuospatial parameters: the digital clock drawing test. JAD.

[39] Poirier G, Ohayon A, Juranville A, Mourey F, Gaveau J (2021). Deterioration, compensation and motor control processes in healthy aging, mild cognitive impairment and Alzheimer's disease. Geriatrics (Basel).

[40] Wild K, Howieson D, Webbe F, Seelye A, Kaye J (2008). Status of computerized cognitive testing in aging: a systematic review. Alzheimers Dement.

[41] Koo BM, Vizer LM (2019). Mobile technology for cognitive assessment of older adults: a scoping review. Innov Aging.

[42] De Roeck EE, De Deyn PP, Dierckx E, Engelborghs S (2019). Brief cognitive screening instruments for early detection of Alzheimer's disease: a systematic review. Alzheimers Res Ther.

[43] Kourtis LC, Regele OB, Wright JM, Jones GB (2019). Digital biomarkers for Alzheimer's disease: the mobile/ wearable devices opportunity. NPJ Digit Med.

[44] Piau A, Wild K, Mattek N, Kaye J (2019). Current state of digital biomarker technologies for real-life, home-based monitoring of cognitive function for mild cognitive impairment to mild Alzheimer disease and implications for clinical care: systematic review. J Med Internet Res.

[45] Hall AO, Shinkawa K, Kosugi A, Takase T, Kobayashi M, Nishimura M, Nemoto M, Watanabe R, Tsukada E, Ota M, Higashi S, Nemoto K, Arai T, Yamada Y (2019). Using tablet-based assessment to characterize speech for individuals with dementia and mild cognitive impairment: preliminary results. AMIA Jt Summits Transl Sci Proc.

[46] Xie H, Wang Y, Tao S, Huang S, Zhang C, Lv Z (2019). Wearable sensor-based daily life walking assessment of gait for distinguishing individuals with amnestic mild cognitive impairment. Front Aging Neurosci.

[47] Varma VR, Ghosal R, Hillel I, Volfson D, Weiss J, Urbanek J, Hausdorff JM, Zipunnikov V, Watts A (2021). Continuous gait monitoring discriminates community-dwelling mild Alzheimer's disease from cognitively normal controls. Alzheimers Dement (N Y).

[48] Polhemus A, Ortiz LD, Brittain G, Chynkiamis N, Salis F, Gaßner H, Gross M, Kirk C, Rossanigo R, Taraldsen K, Balta D, Breuls S, Buttery S, Cardenas G, Endress C, Gugenhan J, Keogh A, Kluge F, Koch S, Micó-Amigo ME, Nerz C, Sieber C, Williams P, Bergquist R, Bosch de Basea M, Buckley E, Hansen C, Mikolaizak AS, Schwickert L, Scott K, Stallforth S, van Uem J, Vereijken B, Cereatti A, Demeyer H, Hopkinson N, Maetzler W, Troosters T, Vogiatzis I, Yarnall A, Becker C, Garcia-Aymerich J, Leocani L, Mazzà C, Rochester L, Sharrack B, Frei A, Puhan M, Mobilise-D (2021). Walking on common ground: a cross-disciplinary scoping review on the clinical utility of digital mobility outcomes. NPJ Digit Med.

[49] Kobayashi M, Kosugi A, Takagi H, Nemoto M, Nemoto K, Arai T, Yamada Y, Lamas D, Loizides F, Nacke L, Petrie H, Winckler M, Zaphiris P (2019). Effects of Age-Related Cognitive Decline on Elderly User Interactions with Voice-Based Dialogue Systems. Human-Computer Interaction – INTERACT 2019. Lecture Notes in Computer Science.

[50] Yamada Y, Shinkawa K, Shimmei K (2020). Atypical repetition in daily conversation on different days for detecting Alzheimer disease: evaluation of phone-call data from regular monitoring service. JMIR Ment Health.

[51] Yamada Y, Shinkawa K, Kobayashi M, Nishimura M, Nemoto M, Tsukada E, Ota M, Nemoto K, Arai T (2021). Tablet-based automatic assessment for early detection of Alzheimer's disease using speech responses to daily life questions. Front Digit Health.

[52] Nasreen S, Rohanian M, Hough J, Purver M (2021). Alzheimer’s dementia recognition from spontaneous speech using disfluency and interactional features. Front. Comput. Sci.

[53] Sisti JA, Christophe B, Seville AR, Garton ALA, Gupta VP, Bandin AJ, Yu Q, Pullman SL (2017). Computerized spiral analysis using the iPad. J Neurosci Methods.

[54] Fellows RP, Dahmen J, Cook D, Schmitter-Edgecombe M (2017). Multicomponent analysis of a digital Trail Making Test. Clin Neuropsychol.

[55] Dahmen J, Cook D, Fellows R, Schmitter-Edgecombe M (2017). An analysis of a digital variant of the Trail Making Test using machine learning techniques. Technol Health Care.

[56] Impedovo D, Pirlo G, Vessio G, Angelillo MT (2019). A handwriting-based protocol for assessing neurodegenerative dementia. Cogn Comput.

[57] Dentamaro V, Impedovo D, Pirlo G (2021). An Analysis of Tasks and Features for Neuro-Degenerative Disease Assessment by Handwriting. Pattern Recognition. ICPR International Workshops and Challenges.

[58] Cilia ND, De Stefano C, Fontanella F, di Freca AS (2021). Handwriting-Based Classifier Combination for Cognitive Impairment Prediction. Pattern Recognition. ICPR International Workshops and Challenges.

